# Structural analysis of the evolution of steroid specificity in the mineralocorticoid and glucocorticoid receptors

**DOI:** 10.1186/1471-2148-7-24

**Published:** 2007-02-16

**Authors:** Michael E Baker, Charlie Chandsawangbhuwana, Noah Ollikainen

**Affiliations:** 1 Department of Medicine, 0693, University of California, San Diego, 9500 Gilman Drive, La Jolla, CA 92093-0693, USA

## Abstract

**Background:**

The glucocorticoid receptor (GR) and mineralocorticoid receptor (MR) evolved from a common ancestor. Still not completely understood is how specificity for glucocorticoids (e.g. cortisol) and mineralocorticoids (e.g. aldosterone) evolved in these receptors.

**Results:**

Our analysis of several vertebrate GRs and MRs in the context of 3D structures of human GR and MR indicates that with the exception of skate GR, a cartilaginous fish, there is a deletion in all GRs, at the position corresponding to Ser-949 in human MR. This deletion occurs in a loop before helix 12, which contains the activation function 2 (AF2) domain, which binds coactivator proteins and influences transcriptional activity of steroids. Unexpectedly, we find that His-950 in human MR, which is conserved in the MR in chimpanzee, orangutan and macaque, is glutamine in all teleost and land vertebrate MRs, including New World monkeys and prosimians.

**Conclusion:**

Evolution of differences in the responses of the GR and MR to corticosteroids involved deletion in the GR of a residue corresponding to Ser-949 in human MR. A mutation corresponding to His-950 in human MR may have been important in physiological changes associated with emergence of Old World monkeys from prosimians.

## Background

The evolution of adrenal and sex steroid signaling has become better understood due to the sequencing of the genes of androgen receptor (AR), estrogen receptor (ER), GR, MR and progesterone receptor (PR) from mammals, amphibia and fish [1-4]. These steroid receptors belong to the nuclear receptor family, a large and diverse family of transcription factors [5]. Sequence analysis of the steroid-binding domains of adrenal and sex steroid receptors reveals that they form a clade that is distinct from other nuclear receptors. The ER is on one branch; the AR, PR, GR and MR, which bind 3-ketosteroids, cluster in another group [3,5,6].

The initial cloning of the MR revealed that its sequence is close to that of the GR [7]. The human GR and MR are about 56% identical in the steroid-binding domain. Moreover, analyses of steroid binding to the MR revealed that cortisol and corticosterone, two glucocorticoids, and aldosterone, the normal physiological mineralocorticoid in mammals, have a similar high affinity for the MR [7-10]. Together these data have led to a consensus that the GR and MR diverged from a common ancestor through gene duplication and divergence [2,3,5]. Recent studies by Bridgham et al. [11] show that the MR is ancestral to the GR.

Still not completely understood is the evolution of steroid specificity in the MR and GR and, in particular, differences between the binding and transcriptional activity of various steroids for the MR. That is, the MR binds progesterone, deoxycorticosterone (DOC), corticosterone, cortisol and aldosterone with a similar high affinity, but only DOC and aldosterone are full agonists of the MR [7-9]. Corticosterone and cortisol have lower transcriptional activity, and progesterone is a mineralocorticoid antagonist.

To investigate these questions, we analyzed the evolution of amino acids at sites that are important in steroid selectivity of vertebrate GRs and MRs in the context of the 3D structures of human GR and MR [12-15]. Our analysis identifies a conserved serine in the MR corresponding to Ser-949 in human MR that is deleted in almost all GRs. This serine is in a loop in the MR that interacts with the D ring in steroids [13-15] and also is close to the AF2-binding domain. This leads us to propose that deletion of this serine was important in evolution of specificity for different corticosteroids in the GR and MR.

Here we also report an unexpected outcome from our sequence analyses: human, chimpanzee, orangutan and macaque MR contain a histidine (His-950 in human MR) that has been absolutely conserved as a glutamine residue in the MR in other mammals including prosimians and New World monkeys, as well as other land vertebrates and fish. Like Ser-949, the Q950H mutation is in a loop that influences the binding of steroids and coactivators [13,16]. The Q950H mutation could alter responses mediated by the MR during the evolution of Old World primates about 35 million years ago, when they and New World monkeys separated from a common ancestor [17].

## Results

### A unique single amino acid deletion in the GR

**Figure 1:**
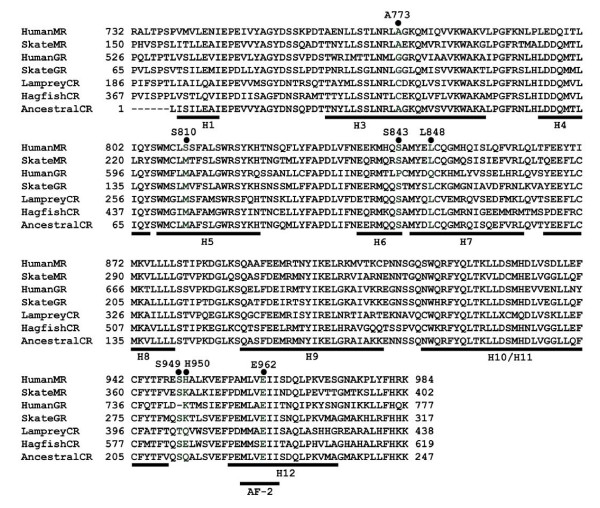
**Alignment of the steroid-binding domain of MR, GR and CR**. Clustal X was used to align the MR, GR and CR sequences. AF2 site is underlined.Pro-637 and Gln-642 in human GR are important in recognition of cortisol [11,15]. Pro-637 and Gln-642 in human GR are conserved in all land vertebrate and teleost GRs. Ser-853 and Leu-848 in human MR are conserved in all land vertebrate MRs, lamprey, hagfish and the proposed ancestral CR and also skate GR.

In Figure 1, we show an alignment of the sequences of human MR and GR with orthologs in skate, a cartilaginous fish, and lamprey and hagfish, two jawless fishes. This alignment reveals that human GR lacks an amino acid corresponding to Ser-949 in human MR. This region on the MR, GR, PR and AR corresponds to the loop between α-helix 11 and α-helix 12 near the C-terminus that positions the AF2 domain for binding to coactivators and corepressors [13,15,16].

**Figure 2:**
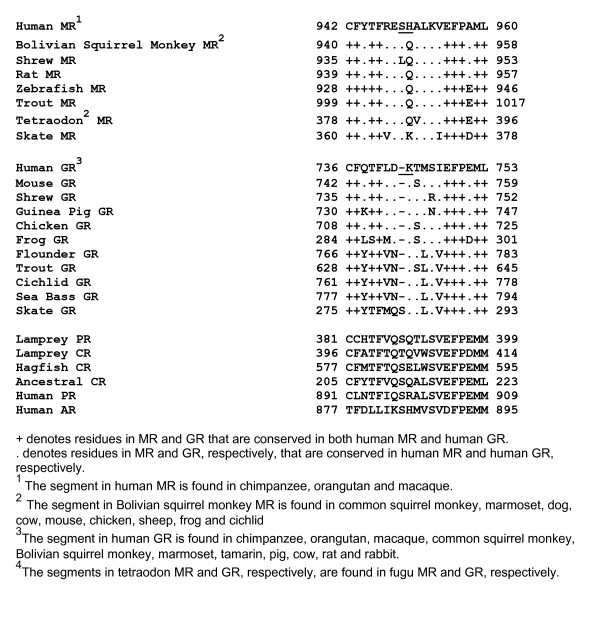
**Alignment of Ser-949 and His-950 in human MR with corresponding residues in other MRs, GRs and human AR and PR**. Ser-949 in human MR is conserved in other MRs. The with the exception of skate GR, all other GRs contain a "signature" deletion of one amino acid, corresponding to Ser-949 in human MR. His-950 in human MR is conserved in human MR and other Old World primates. A glutamine is found in other MRs in land vertebrates and teleosts at this site. A corresponding lysine and glutamic acid and lysine are found in skate MR and hagfish CR, respectively. A corresponding glutamine is found in lamprey PR and CR.

Analysis of a more extensive collection of GRs and MRs in Genbank revealed that almost all GRs lack an amino acid at this position in the MR. The only exception is the skate GR, which contains a serine [Figure 1]. Figure 2 shows the region around human MR Ser-949 in vertebrate MRs and GRs, human AR and PR, lamprey PR and corticosteroid receptor (CR) and hagfish CR. With the exception of skate GR, the other GRs contain a "signature" deletion of one amino acid at the position corresponding to Ser-949 in the MR.

Figure 2 also shows that Ser-949 in human MR is highly conserved. With the exception of tree shrew MR, the other MRs contain a serine at this position. Lamprey CR contains a threonine, which is a conservative replacement of serine. The presence of serine and threonine at this site in hagfish CR and lamprey CR, respectively, is especially important because lamprey and hagfish are jawless fishes at the base of the vertebrate line. Indeed, Bridgham et al. [11] predicted that the ancestral CR has this serine.

**Figure 3:**
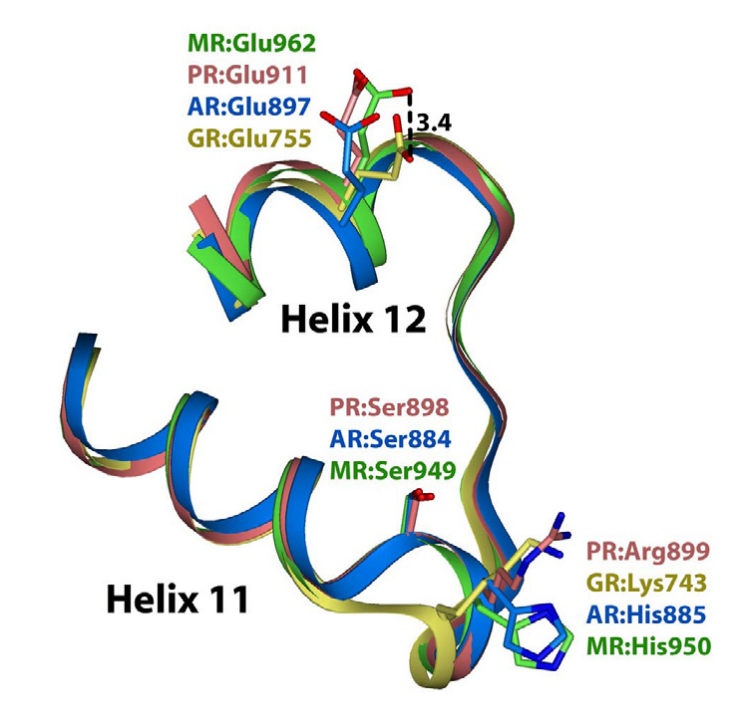
**Comparison of the 3D structure of the region containing Ser-949 and His-950 in human MR with other steroid receptors**. Ser-949 in human MR and corresponding serine residues in human PR and AR are close to each other and have the Oγ side chain directed away from the receptor core. Human GR lacks this loop. The side chain of Lys-743 in the GR is displaced from His-950 in the MR. In the AF2 binding-domain, Oε2 on Glu-755 in the GR is displaced 3.4 A from Oε2 on Glu-962 in the MR.

The recently reported 3D structures of the MR [13-15] permit an analysis of the interactions between amino acids and steroids in the region containing the loop joining helix 11 and helix 12 in the MR and its comparison with the GR [12], PR [18] and AR [19-21]. As shown in Figure 3, Ser-949 in the MR aligns nicely with the corresponding serine in the PR and AR. As expected, the serine deletion in the GR leads to a different conformation in this part of the loop. It also may contribute to a different orientation in α-helix 12 of Glu-962 in the MR and Glu-755 in the GR [Figure 3, see Additional file 1].

**Figure 4:**
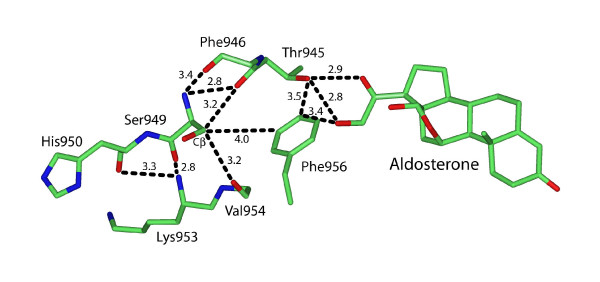
**Interaction of Ser-949 and His-950 with other residues in human MR**. The Ser-949 backbone oxygen and nitrogen form hydrogen bonds with backbone oxygen and nitrogen on nearby residues. Ser-949 Cβ has a van der Waals interaction with Phe-946, Val-954 and Phe-956. The side chain on His-950 is pointed away from the MR core and could interact with proteins that bind to the MR.

**Figure 5:**
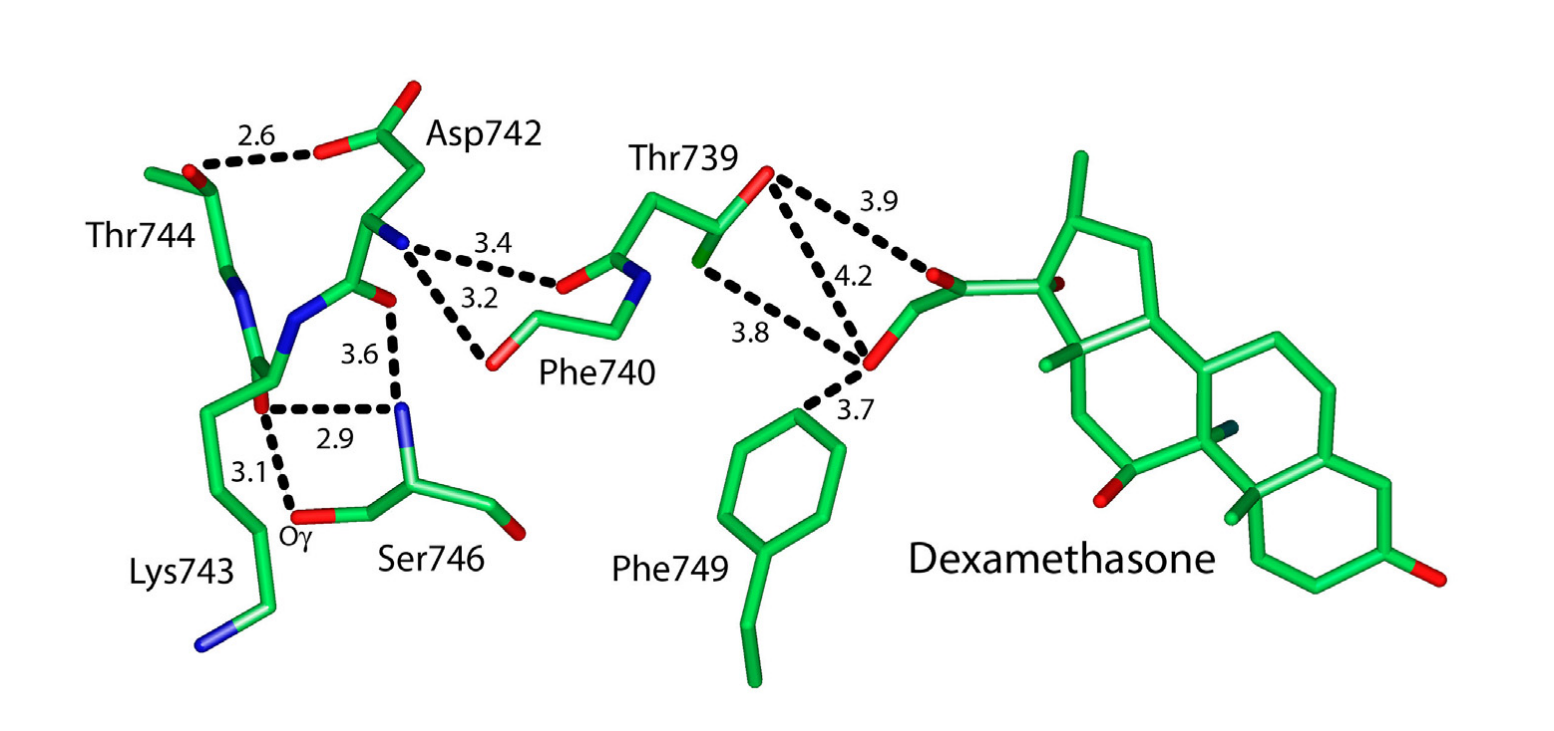
**Interaction of Asp-742 with other residues on the GR**. The Asp-742 backbone oxygen and nitrogen form hydrogen bonds with backbone oxygen and nitrogen on nearby residues. Asp-742 also forms a hydrogen bond with Oγ1 on Thr-744. The side chain on Lys-743 is pointed away from the GR core and could interact with proteins that bind to the GR.

In the human MR, Ser-949 has stabilizing interactions with Thr-945, Phe-946, Arg-947, Glu-948, His-950, Ala-951, Leu-952, Lys-953, Val-954, some of which are shown in Figure 4. Most of these interactions involve hydrogen bonds between the backbone oxygen and nitrogen on Ser-949 with backbone atoms on nearby residues. Also of interest is the van der Waals interaction between Cβ on Ser-949 with Cε1 on Phe-956, which is close to the C21-hydroxyl on aldosterone and deoxycorticosterone [13,14]. A corresponding phenylalanine group also stabilizes steroid binding in the GR [Figure 5], PR and AR.

Asp-742 in human GR aligns with Ala-951 in human MR. However, Asp-742 in the GR appears to have some of the stabilizing interactions of Ser-949 in the MR [Figure 5]. There are numerous hydrogen bonds between the backbone oxygen and nitrogen of Asp-742 and those on nearby residues. An important difference between Asp-742 and Ser-949 is that Oδ2 on Asp-742 interacts with Oγ1 on Thr-744.

### A unique mutation in human, chimpanzee, orangutan and macaque MR

Human, chimpanzee, orangutan and macaque MR contain His-950 [Figure 2], instead of glutamine as found in the MR in New World monkeys, prosimians and other available MRs. Moreover, lamprey CR and the proposed ancestral CR [11] have a glutamine; hagfish CR has a glutamic acid at this site. These data indicate the mutation from glutamine to histidine occurred relatively late in the evolution of the MR.

Interestingly, human AR has a corresponding His-885, which is found in mammalian AR but not in amphibian AR, which has a glutamine at this position [data not shown]. As seen in Figure 3, MR His-950, PR Arg-899, GR Lys-743 and AR His 885 are oriented away from the steroid binding site and are in a position to interact with other proteins.

## Discussion

The MR is ancestral to the GR [11,22]. This pathway for the divergence of the MR and GR from a common ancestor provides a context for examining data on single and double mutations that change the activation of the GR and MR by various steroids [11,15,23,24] and analyses of functionally important sites in the GR and MR [Figures 3 and 4] in order to understand the evolution of transcriptional specificity for corticosteroids in the MR and GR.

### Role of helix 3 and helix 5 in MR steroid specificity

One of the most exciting discoveries from a clinical and evolutionary perspective was the identification of a human MR with a S810L mutation, in which progesterone is mineralocorticoid agonist instead of an antagonist [23]. At a concentration of 1 nM, progesterone, 19-nor progesterone, 17α-hydroxyprogesterone and pregnenolone significantly activate the S810L mutant MR. Geller et al. [23] constructed a 3D model of the L810 mutant MR, which indicated that its increased activity with progestins is due to a stabilizing van der Waals interaction between to Leu-810 on helix 5 and Ala-773 on helix 3. Mutations at residues 810 and 773, changed the distance between side chains and altered transcriptional activation of human MR by 19-nor-progesterone.

3D structure of human MR810L mutant complexed with progesterone revealed that Leu-810 and Ala-773 are separated by 4.25A to 4.5A, which is a weak van der Waals interaction [13,14]. However, the 3D structure shows that Leu-810 and Ala-773 have hydrophobic interactions with the C19 methyl group on progesterone. Moreover, Leu-810 has stabilizing interactions with other amino acids in helix 3 [13,14].

The data from Geller et al. [23], Bledsoe et al. [13] and Fagart et al. [14] show that a single mutation in the MR yields a receptor that is activated by steroids, such as progesterone and pregnenolone, which lack the C21 hydroxyl substituent. This provides a mechanism for loss of activation of an ancestral MR by progesterone.

Interestingly, later studies showed that a single mutation at the corresponding residues in helix 3 and helix 5 in the GR and PR can change activation of the GR by glucocorticoids and mineralocorticoids and the PR by progestins [24].

### GR activation by 17α-hydroxy-steroids

The crystal structure of human GR with dexamethasone [12] identified a small pocket containing Pro-637 and Gln-642, which was unique to the GR, and that Gln-642, interacted with the 17α-hydroxyl group on dexamethasone. Pro-637 and Gln-642 on human GR aligns with Ser-843 and Leu-848 in the human MR, which are conserved in all available MR sequences and also in lamprey, hagfish and the ancestral CR, as well as, skate GR [Figure 1]. Pro-637 and Gln-642 are conserved in the other GR sequences [Figure 1]. Bledsoe et al. noted that the hydrophobic side chain in Leu-848 in human MR would not have a favorable stabilizing interaction with the 17α-hydroxyl group on cortisol and dexamethasone.

Using the 3D structures of human MR and GR for guidance, Li et al. [15] investigated the roles of Ser-843 and Leu-848 in human MR and Pro-637 and Gln-642 in human GR in the response to cortisol and corticosterone. They mutated Ser843 and Leu848 in human MR to proline and glutamine, respectively. The mutant MR had an increased response to cortisol. They also mutated Pro-637 and Gln-642 in human GR to serine and leucine, respectively. These MR-like mutations in the GR reduced its transcriptional response to cortisol compared to that of corticosterone, which lacks a 17α-hydroxyl group. This indicated that Pro-637 and Gln-642 were important in the response of the GR to 17α-hydroxy-glucocorticoids.

Bridgham et al. [11] performed similar experiments with the proposed ancestral CR comparing the activity of aldosterone, cortisol and DOC with different mutant ancestral CRs. Bridgham et al. [11] found that a single mutation of CR Ser-106, which corresponds to human MR Ser-843 [Figure 1], to proline increased the EC50 of aldosterone from 0.23 nM to 70 nM, cortisol from 5.7 nM to >1000 nM and DOC from about 0.23 nM to about 15 nM. However, adding a second L111Q mutation to form the S106P/L111Q ancestral CR lowers the EC50 of cortisol to 72 nM. This is consistent with a stabilizing interaction between Gln-111 and the 17α-hydroxyl group on cortisol. Also, the EC50 of aldosterone for S106P/L111Q ancestral CR increases to 148 nM and that of DOC remains at about 15 nM. Thus, the CR S106P/L111Q mutant was more like the GR. The absence of Pro-106 and Gln-111 in the ancestral CR and in the corresponding residues in skate GR can explain the reduced activity of cortisol and the preference of these receptors for aldosterone and DOC. Bridgham et al. [11] proposed that specificity for glucocorticoids arose in a descendent of an elasmobranch.

An important difference between the human MR and ancestral CR is that the human L848Q MR mutant retains its activity for cortisol and only has a 10 fold loss in activity for corticosterone [15], while ancestral L111Q ancestral CR mutant loses most of its activity towards cortisol, aldosterone and DOC [11]. This indicates that although the Ser/Pro and Leu/Gln mutations are important for-specificity for 17α-hydroxy-steroids, other amino acids influence the role of Leu and Gln on the MR and GR, respectively, in this response.

### A conserved serine in the MR that is deleted in the GR

We propose that a conserved serine corresponding to Ser-949 in human MR [Figures 2 and 3] also contributes to the differences in steroid specificity between the MR and GR. Vertebrate GRs, with the exception of skate GR, contain a unique deletion at the position corresponding to Ser-949 in human MR [Figures 2 and 3], which indicates that this deletion occurred in a descendent of the skate GR.

The presence of this serine in skate GR [Figure 1] is important because Bridgham et al [11] find that aldosterone and cortisol have transcriptional activity for skate GR that resembles that of human and other tetrapod MRs and differs from the steroid specificity of tetrapod GRs. The EC50 of aldosterone for skate GR is over 10-fold lower than that of cortisol [11], which is reversed in tetrapod GR [8,9,11,25,26]. We propose that deletion of Ser-282 in skate GR, which is conserved in all descendent GRs was important in the evolution of GR.

### Ser-949 is in a loop that positions the AF2 domain

A role for Ser-949 in the response to steroids is reasonable because Ser-949 is at the beginning of the loop involved in positioning of the AF2 domain for productive interaction with coactivators after the receptor binds steroids [27,28]. This loop in the MR and GR also interacts with the D ring of steroids [12-15] [Figures 4 and 5]. Binding of antagonists or partial agonists to steroid receptors prevents positioning of AF2 for optimal interaction with coactivators. Analysis of the 3D structure of the MR [13] confirms the importance of the loop between helix 11 and helix 12 in promoting optimal positioning of AF2. The 3D structure of human MR Glu-962 and GR-755 reveals that they have different orientatations [Figure 3, see Additional file 1].

Moreover, there is evidence that mutations in the loop positioning AF2 can alter transcriptional activity of the MR, even when the mutant MR retains high affinity for the steroid [16,25,26]. Mutagenesis studies by Hellal-Levy *et al*. [16] show that the loop between helix 11 and helix 12 in human MR is important in transcriptional activation by aldosterone. Aldosterone has high affinity for MR mutants K953A and V954A. However, in the presence of aldosterone, these MR mutants had little transcriptional activity and did not bind certain coactivators. Lys-953 and Val-954 have stabilizing interactions with Ser-949 [Figure 4].

Also Hultman *et al*. [25] showed that mutation in AF2 of a highly conserved Glu-962 to alanine in human MR altered the transcriptional activity of aldosterone and cortisol, although the binding of these two steroids to the MR was unchanged [26]. They found that aldosterone retained its activity as an agonist, although with reduced potency. In contrast, cortisol changed from a partial agonist to an antagonist [25].

### Implications for the evolution of steroid specificity for MR and GR

Bridgham et al. [11] provide strong evidence that a GR with a preference for cortisol over aldosterone arose in an elasmobranch descendent that was the common ancestor of ray finned fish and land vertebrates. We propose that an important contribution to this change was a deletion corresponding to Ser-282 in skate GR, which would be expected to change the orientation of the AF2 domain in descendent GRs and affect binding to coactivators [13,15,22].

**Figure 6:**
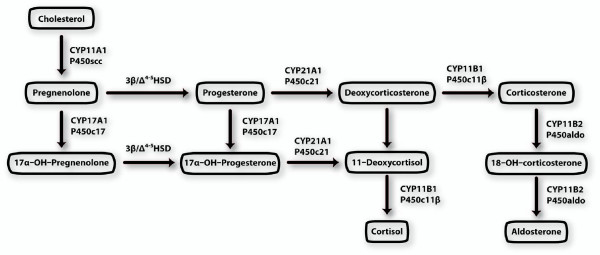
**Pathways for synthesis of adrenal steroids**. Aldosterone is at the end of the pathway, in contrast to progesterone and deoxycorticorticosterone, which are near the beginning.

The identity of the steroid(s) that activate the ancestral CR and hagfish and lamprey CR has yet to be determined. Bridgham et al. find that transcription by hagfish CR is stimulated 35- to 45-fold in the presence of either 100 nM cortisol, corticosterone, DOC or aldosterone. Transcription by hagfish and lamprey CR is stimulated by about 10-fold and 5-fold, respectively, in the presence of 100 nM progesterone. Active steroids in lamprey appear to have 15α-hydroxyl-substituents such as 15α-OH-estradiol, 15α-OH-testosterone and 15α-OH-progesterone [29-32]. Bridgham et al. did not study the activity of 15α-OH-progesterone. Nevertheless, their data suggest that DOC and/or progesterone or one of their hydroxylated derivatives were among the ligands for the ancestral CR. This supports an earlier hypothesis based on the pathway for synthesis of adrenal steroids from cholesterol [Figure 6] that DOC was a ligand for the ancestral CR because its synthesis is simpler than that corticosterone, cortisol and aldosterone [4,33,34].

### DOC is not metabolized by 11β-hydroxysteroid dehydrogenase

In land vertebrates, 11β-hydroxysteroid dehydrogenase-type 2 (11β-HSD2) has an important role in aldosterone activation of the MR [35-37]. The C11-hydroxyl on aldosterone is inert to 11β-HSD2 due to the formation of a complex between the C11-hydroxyl and the C18-aldehyde. On the other hand, the C11-hydroxyl on cortisol and corticosterone is readily oxidized to a ketone, yielding cortisone and 11-dehydrocorticosterone, which are inactive steroids. Thus, in the presence of excess cortisol or corticosterone, aldosterone can activate the MR in the distal tubule of the kidney and in other tissues that contain 11β-HSD2.

DOC lacks a C11-hydroxyl group and would not be metabolized by 11β-HSD2. In this respect, DOC resembles aldosterone. DOC and corticosterone are more active than cortisol when binding to hagfish and lamprey CR. The EC50s of DOC and corticosterone for activation of hagfish and lamprey CR [11] are at least 10-fold lower than that of cortisol. In hagfish, lamprey and skate, DOC could occupy the CR and MR in the tissues in which 11β-HSD2 was expressed, while corticosterone and cortisol would be metabolized to inactive steroids, Later, in lungfish and land vertebrates, aldosterone joined DOC as a second steroid that could regulate the mineralocorticoid response in the presence of 11β-HSD2 [4,34].

### A role for the MR in the evolution of Old World primates?

We propose that conservation of glutamine in the MR in fish and most land vertebrates including prosimians and New World monkeys at the position that corresponds to His-950 in the human MR indicates that this glutamine is important. At this time, it is not known if the Q950H mutation in Old World primates is functionally important. Supporting a function for the Q950H mutation is the different chemistry of histidine, which has a side chain with a pKa of about 7, unlike glutamine. Histidine and glutamine also differ in the spatial characteristics of their side chains. The location of His-950 and Gln-950 at the beginning of the loop that influences the position of the AF2 domain supports functional importance.

The 3D structure of the MR [13-15] shows that the His-950 side chain points away from the core of the MR [Figure 3]. This would facilitate binding to coregulator proteins, which is important because differential cellular levels of coactivators and corepressors can regulate tissue-specific actions of estrogens [38], progestins [39] and corticosterone activation of the GR and MR [40]. The Q950H mutation in the MR in Old World monkeys may have altered the interaction of the MR with one or more coregulators in specific tissues, influencing the evolution of physiological responses to aldosterone and cortisol in Old World monkeys.

It needs be noted that Hellal-Levy *et al*. [16] found that the H950A MR mutant is fully active in the presence of aldosterone. However, they did not study the effect of DOC, cortisol, progesterone or other steroids on the transcriptional activity of the H950A mutant or of an H950Q mutant. Nor did they study transcriptional activity in the presence of a variety of coactivators and corepressors that Li *et al*. [15] and Hultman *et al*. [25] studied. This is important because the evolution of the His-950 MR is likely to have subtle physiological effects as humans retain the MR functions found in mammals that have the Gln-950 MR. Comparative studies using wild-type and H950Q mutant human MR with various agonists, partial agonists and antagonists in the presence of different coactivators and corepressors are needed to elucidate the function of His-950.

## Conclusion

A deletion corresponding to Ser-282 in skate GR was important in the evolution of different responses to corticosteroids by the MR and GR. DOC and progesterone or their hydroxylated derivatives are candidate steroids for activation of the ancestral CR in hagfish and lamprey. A mutation in the MR at a position corresponding to His-950 may have been important in the emergence of Old World monkeys.

## Methods

The steroid-binding domains of human GR and MR were used for BLAST [41] searches of GenBank and Ensembl to extract their vertebrate orthologs and other PR AR and CR sequences. The steroid-binding domains were aligned with ClustalX [42]. 3D structures were taken from the Protein Data Bank (PDB). For our analyses, we used PDB files of the MR [PDB:2AA2], GR [PDB: 1M2Z], PR [PDB: 2A28] and AR [PDB: 1I37].

## Authors' contributions

MEB conceived of this project, drafted the manuscript and supervised the research. CC and NO carried out BLAST searches, sequence alignments, structural analysis and preparation of the 3D figures. All authors have read and approved the final manuscript.

## Supplementary Material

Additional File 1 3D structure of the conserved glutamic acid in the AF2 domain. The conserved glutamic acid in the AF2 domain in α-helix 12 on the GR has a different orientation than the corresponding glutamic acid in the MR, PR and AR. Oε2 on the GR Glu-755 is 3.4 A from Oε2 on MR Glu-962. Click here for file
