# Supplementary figures and images for: Structural analysis of the evolution of steroid specificity in the mineralocorticoid and glucocorticoid receptors
Source: BMC Evol Biol. 2007 Feb 16;7:24. doi: 10.1186/1471-2148-7-24 (PMC1805736; doi:10.1186/1471-2148-7-24)

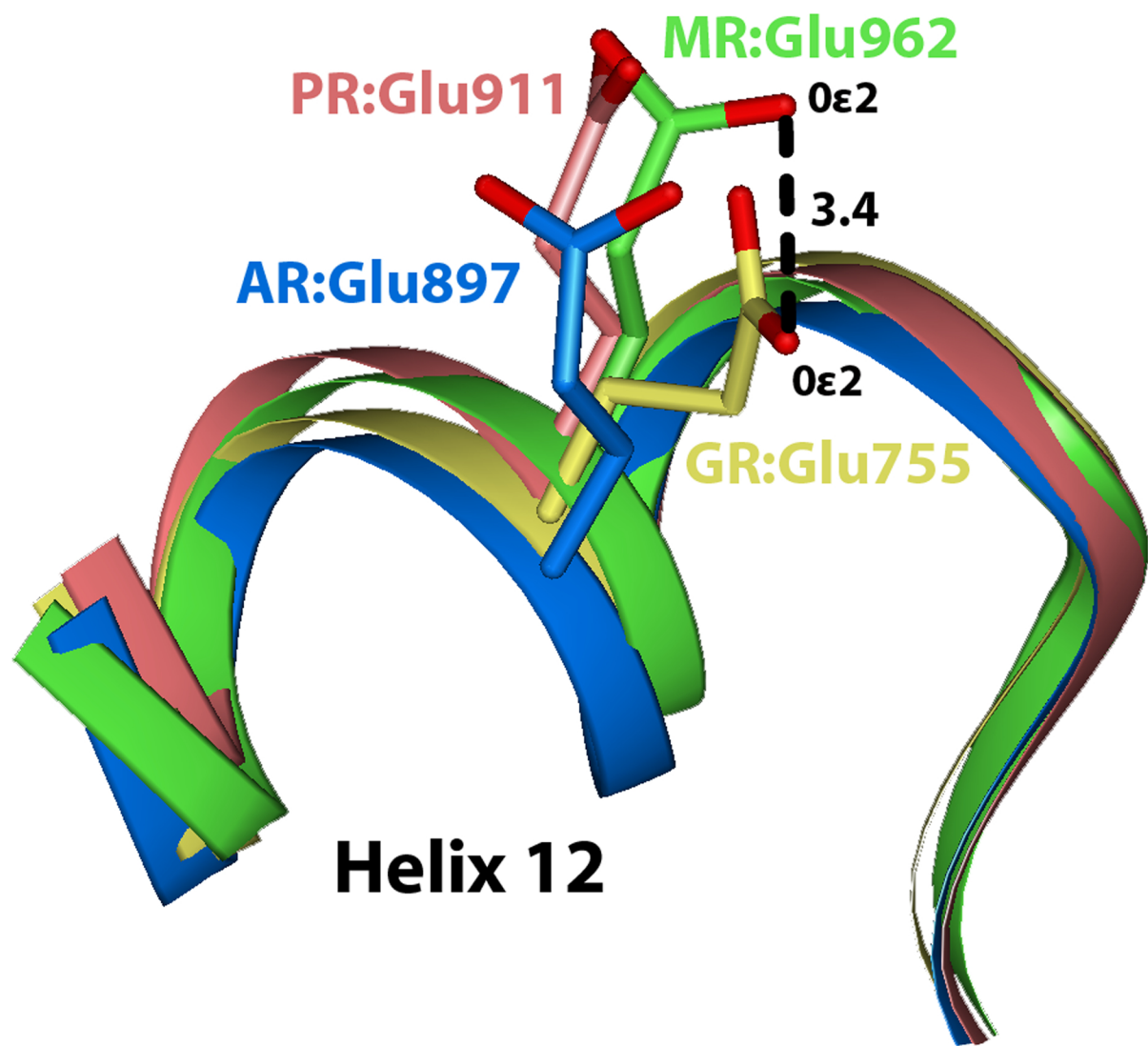

Supplement: Additional File 1 — 3D structure of the conserved glutamic acid in the AF2 domain. The conserved glutamic acid in the AF2 domain in α-helix 12 on the GR has a different orientation than the corresponding glutamic acid in the MR, PR and AR. Oε2 on the GR Glu-755 is 3.4 A from Oε2 on MR Glu-962. [file 1471-2148-7-24-S1.pdf]
